# Botulinum Toxin: Preclinical and Clinical Aspects

**DOI:** 10.1007/s00702-026-03145-9

**Published:** 2026-04-01

**Authors:** Dirk Dressler, Jürgen Frevert

**Affiliations:** 1 Movement Disorders Section, Department of Neurology, Hannover Medical School, Carl-Neuberg-Str. 1, D-30625 Hannover, Germany; 2 Botulinum Toxin Research Centre, Tongji University Medical School, Shanghai, China; 3 Merz Pharmaceuticals, Frankfurt/M, Germany

Hans Bigalke died on 10 October 2024. Once the initial shock had eased, Jürgen and I decided to edit a selection of manuscripts in honour of our dear friend. These manuscripts should deal with botulinum toxin, the central theme of Hans's work and life. They should cover aspects of botulinum toxin research Hans was involved with and they should be contributed to by authors who were close to him. We soon came up with a wish list and were overwhelmed, when almost all of the authors we invited readily accepted our invitation. Best of all, they really delivered in the end, albeit with the minor delays natural. As always, reviewing and revisions took their time. Now, at the beginning of this new year of 2026, everything is ready.

We hope we could pull together a couple of manuscripts fulfilling our initial criteria and, first and foremost, are interesting and enlightening to the readers of this journal.

The first manuscript discusses the properties of a botulinum neurotoxin-like protein from enterococcus which cleaves SNAP23, suggesting that the botulinum toxin induced excretion block of acetylcholine in neuronal cells might be a model to block the secretion of other substances in other cells.

The second manuscript covers the structural aspects of botulinum toxins that are crucial for their action as biologics.

The third manuscript is a personal account of the challenges involved in ‘taming the beast’, manufacturing the first botulinum toxin for therapeutic purposes.

The fourth manuscript is a review of the mouse diaphragm assay developed by Hans to detect botulinum toxin antibodies, determine the potency of botulinum toxin preparations and to perform a variety of translational studies on botulinum toxins.

The fifth manuscript addresses the primary concern that botulinum toxin therapy faced, when it was proposed as a treatment.

The sixth manuscript describes the many challenges of developing botulinum toxin drugs, challenges known and challenges unexpected.

The seventh manuscript reports work already started with Hans’s input. It gives an overview about about currently available botulinum toxin drugs.

The eighth manuscript takes us to the Korean botulinum toxin manufacturing cluster, which is now the most prolific one in the world.

The ninth and final manuscript documents the use of botulinum toxin in pain treatment, a huge field in medicine and a highly lucrative market for botulinum toxin manufacturers.

